# Rickets Secondary to Distal Renal Tubular Acidosis: A Rare Etiology With a Five-Year Follow-Up of Clinical and Radiological Outcomes and Literature Review

**DOI:** 10.7759/cureus.78454

**Published:** 2025-02-03

**Authors:** Hiba Nasar, Imad Nasar, Maryam G Ansari, Ariba Nasar, Ahmad G Ansari

**Affiliations:** 1 Department of Pediatrics, Jawaharlal Nehru Medical College, Aligarh Muslim University, Aligarh, IND; 2 Department of Orthopedics, RAK Medical and Health Sciences University, Ras Al Khaimah, ARE; 3 Department of Pathology, Baba Raghav Das Medical College, Gorakhpur, IND; 4 Department of Medicine, Jawaharlal Nehru Medical College, Aligarh Muslim University, Aligarh, IND; 5 Rajiv Gandhi Centre for Diabetes and Endocrinology, Jawaharlal Nehru Medical College, Aligarh Muslim University, Aligarh, IND

**Keywords:** distal renal tubular acidosis, growth disorders, nephrocalcinosis, non-anion gap metabolic acidosis, rickets

## Abstract

Distal renal tubular acidosis (dRTA) is one of the rare causes of rickets in children. In this case report, we describe a 13-year-old male patient who presented with short stature, bilateral genu valgum, and a history of recurrent weakness in the lower limbs. Radiological observations were consistent with rickets with bony deformities. Detailed evaluation revealed normal serum vitamin D levels and normal anion gap metabolic acidosis with hypokalemia and hyperchloremia. Subsequent investigations led to the diagnosis of dRTA (type 1). The patient was treated with alkali therapy and potassium supplementation and was monitored over a five-year period, during which progressive improvement in clinical manifestations and radiological and biochemical parameters were observed. This case report underscores the importance of systematic assessment of nonnutritional causes of rickets in pediatric patients. Early diagnosis and treatment are crucial to prevent long-term complications, including growth failure and bone deformities.

## Introduction

Rickets, a disorder characterized by impaired mineralization of the bone matrix, is a common cause of skeletal deformities and growth retardation in children [1]. Nutritional rickets, primarily caused by vitamin D deficiency, remains the most prevalent form and is often associated with inadequate sunlight exposure, poor dietary intake, or malabsorption [2]. Rickets is further classified into calcipenic, i.e., due to calcium deficiency, vitamin D deficiency or resistance, genetic or drug induced; or phosphopenic, i.e., due to deficiency of phosphate, usually due to impaired intestinal phosphate absorption or abnormal renal excretion of phosphate [3]. Distal renal tubular acidosis (dRTA) is one of the renal tubular disorders, which is characterized by the inability of the distal tubules to acidify urine appropriately, resulting in metabolic acidosis, rickets-like skeletal abnormalities, and renal stones [4]. This metabolic disturbance interferes with bone mineralization and contributes to skeletal deformities such as genu valgum. In this case report, we describe an approach to a patient who presented with a nonnutritional etiology of rickets.

## Case presentation

**Figure 1:**
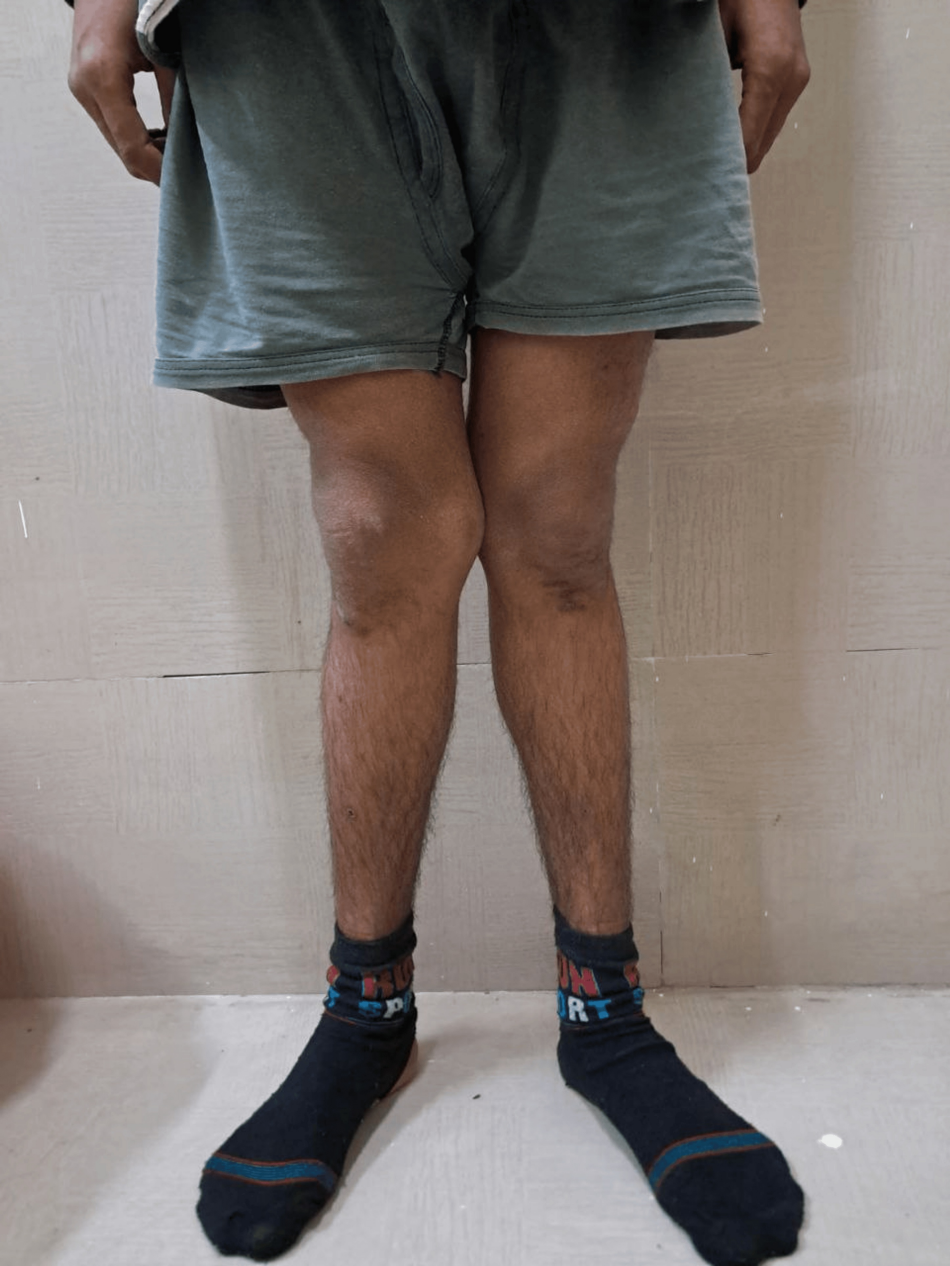
Clinical image of the patient showing genu valgum

**Figure 2:**
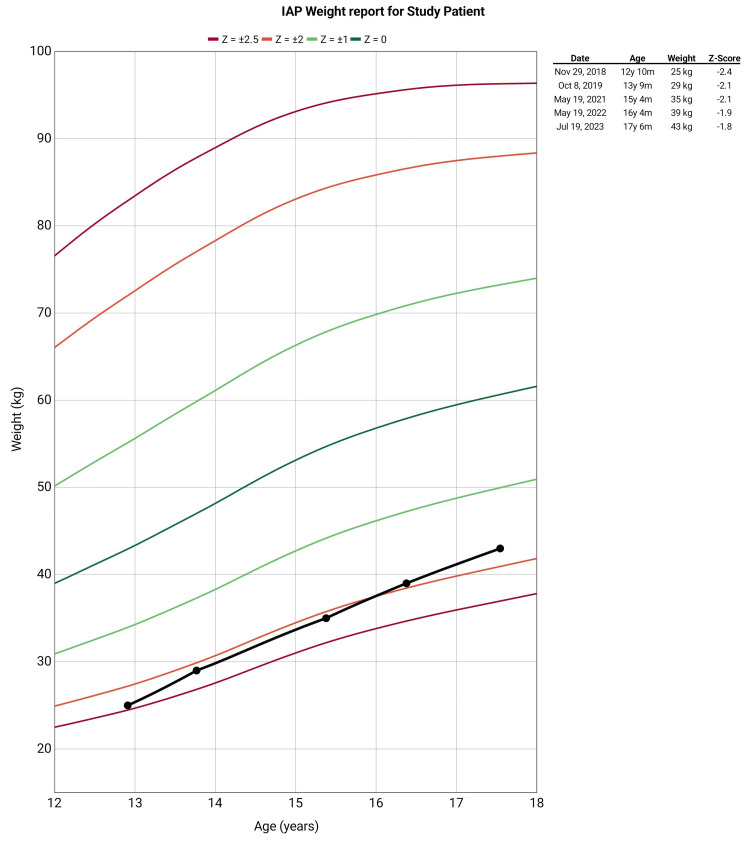
Weight for age of the study patient plotted according to the Indian Academy of Pediatrics (IAP) growth charts Data entry done using the Child Growth Tracker android application, *ABQ App source, LLC,* and the chart was plotted according to the Revised IAP growth charts

**Figure 3:**
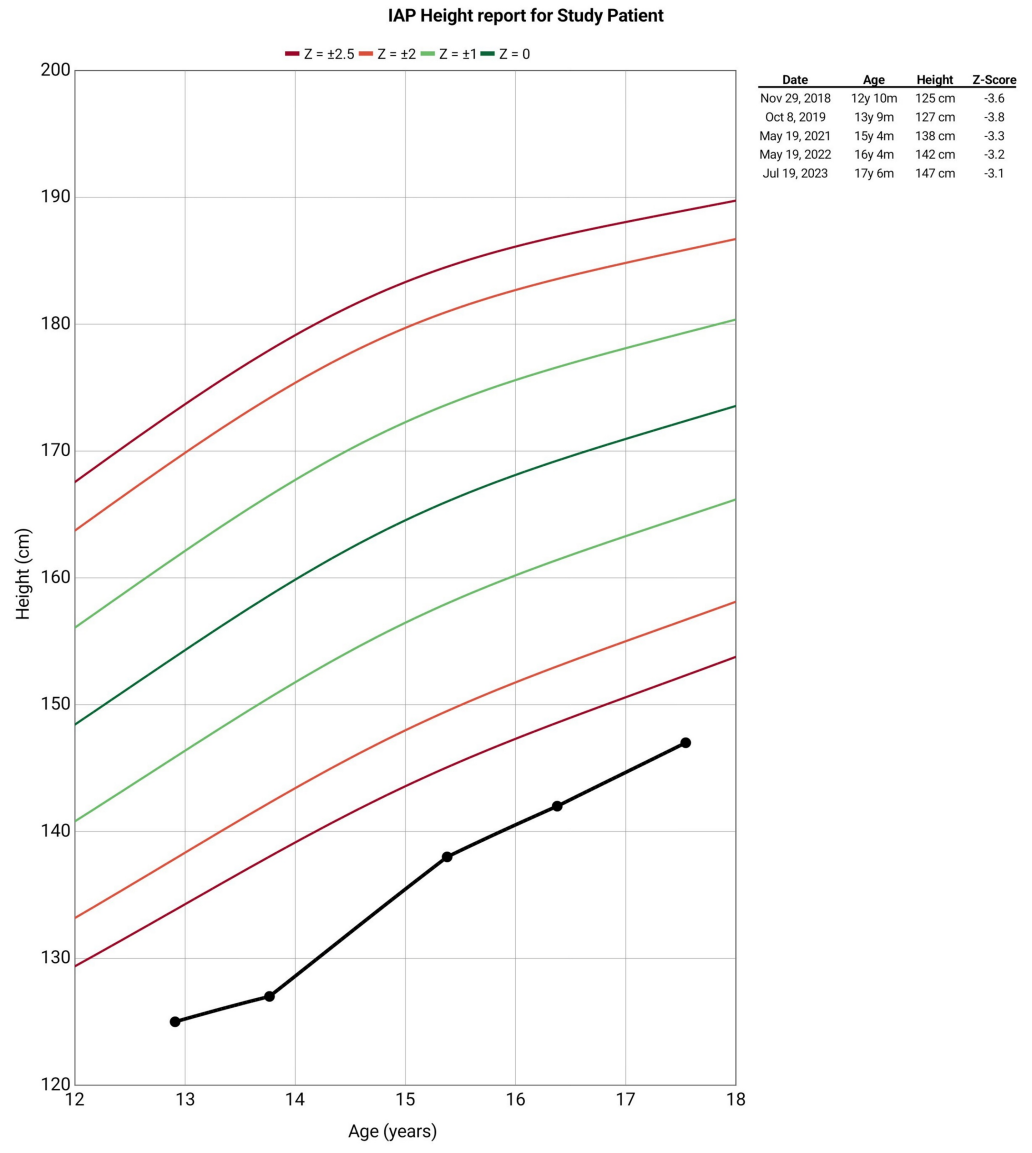
Height for age of study patient plotted according to Indian Academy of Pediatrics (IAP) growth charts Data entry was done using the Child Growth Tracker android application, *ABQ App Source, LLC,* and the chart was plotted according to the Revised IAP growth charts

A 13-year-old male patient presented to our outpatient department with a complaint of deformities in bilateral lower limbs which has progressed over a period of seven years. The patient also reported a history of fatigue, bone pain, muscle cramps, and history of intermitted acute onset weakness which resolved completely after visiting to local medical practitioner (no records were available with the patient). The patient developed these symptoms over the past six years, and the complaints were progressive. There was no history of poor nutritional intake, failure to thrive, delayed milestone development, or similar deformities in siblings as reported by the parents. There was no history suggestive of urinary tract infections, hematuria, excessive thirst or polyuria, chronic diarrhea, fat malabsorption or steatorrhea, prolonged vomiting, or prior gastrointestinal surgery. There was no history suggestive of any systemic or chronic illness. There was also no history suggestive of fragility fractures. On examination, the patient was lean-built, and the physical examination revealed bilateral genu valgum with no other significant findings (Figure 1). Anthropometric data at presentation revealed short stature and low weight for age (height: 125 cm, with Z score: -3.6; and weight: 25 kg, with Z score -2.4 as per the Revised Indian Academy of Pediatrics Growth Charts) (Figures 2, 3) [5].

**Figure 4:**
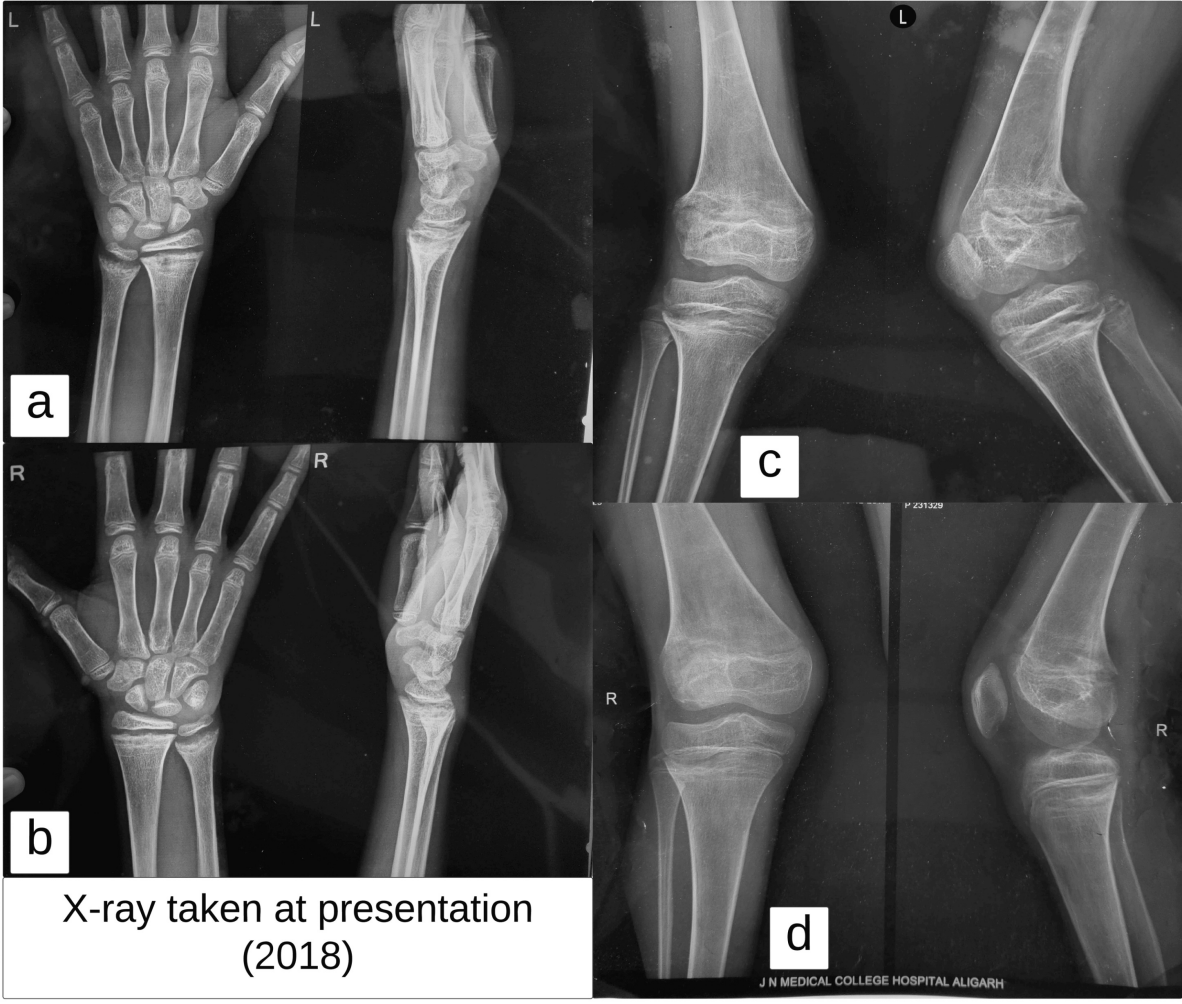
(a) and (b): X-ray bilateral hand with wrist showing widening, fraying, and cupping of the metaphyseal ends of the distal radius and ulna; prominent widening of the growth plates and generalized osteopenia; (c) and (d): X-ray bilateral knee showing bilateral genu valgum deformity with widening and cupping of the metaphyseal ends of the femur and tibia, widened and irregular growth plates, and reduction in the cortical thickness of the long bones

**Figure 5:**
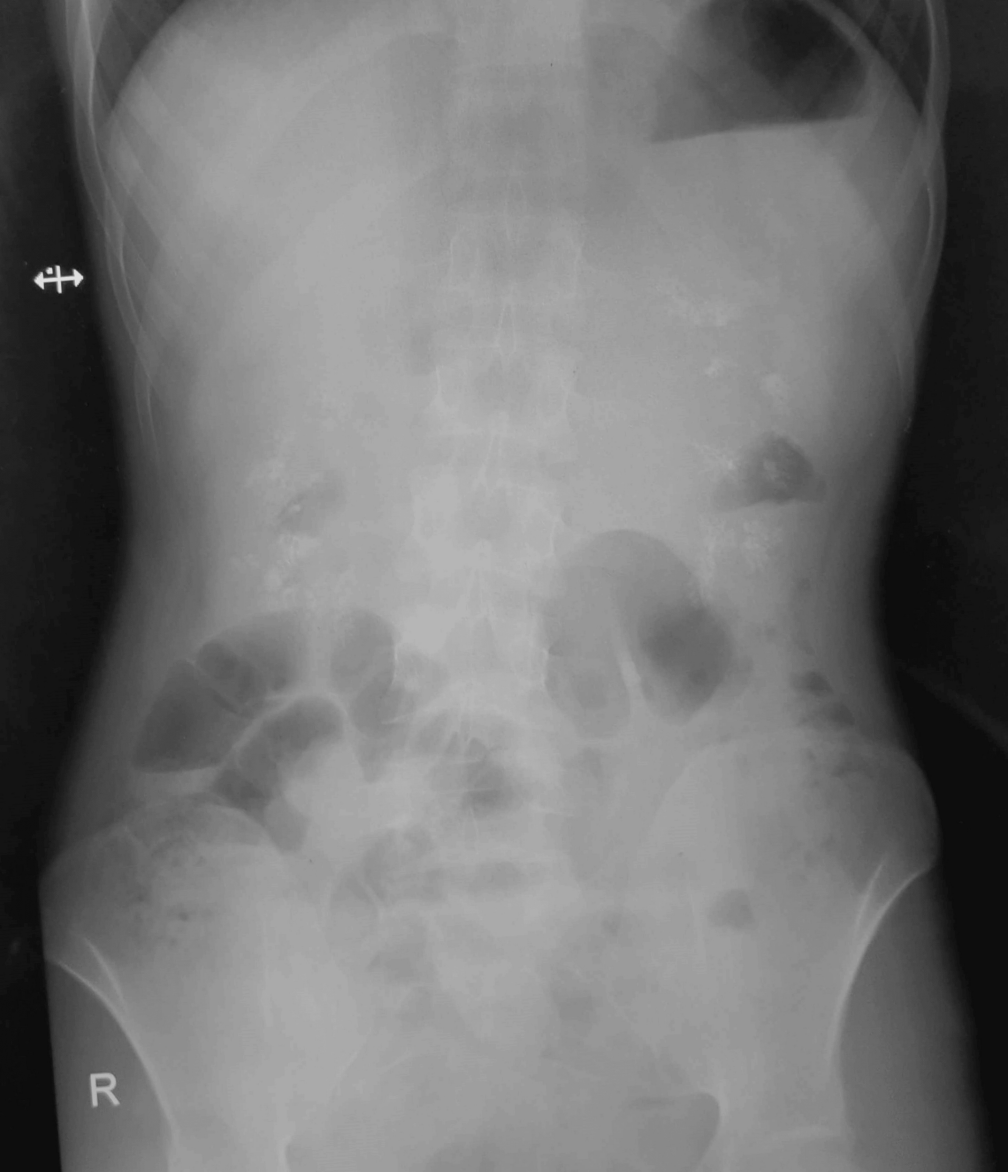
X-ray abdomen anteroposterior (AP) view of the patient showing diffuse increase in radiodensity observed in the bilateral kidneys consistent with nephrocalcinosis with no evidence of calculi in the ureters or urinary bladder or radiographic signs of urinary tract obstruction

X-ray of the bilateral lower limbs was performed and showed bilateral genu valgum deformity with widening and cupping of the metaphyseal ends of the femur and tibia, widened and irregular growth plates, and reduced cortical thickness of the long bones. X-ray of the bilateral hand with wrist joint showed widening, fraying, and cupping of the metaphyseal ends of the distal radius and ulna; prominent widening of the growth plates; and generalized osteopenia (Figure 4). These findings suggested rickets as our primary differential diagnosis. Laboratory and biochemical analyses were done and are shown in Table 1. Initial workup revealed normal serum calcium, phosphorus, alkaline phosphatase, Vitamin D, and intact parathyroid hormone levels. These findings suggested etiology other than the common nutritional deficiency rickets and further workup was performed which revealed normal anion gap metabolic acidosis with hyperchloremia and hypokalemia. Urine analysis showed urinary pH of 8 and 24 urinary calcium values of 465 mg/day. The urine anion gap was elevated, and the fractional excretion of bicarbonate was 3.8% (Table 2). The patient’s ultrasound abdomen was performed and showed bilateral nephrocalcinosis which was also evident in the patient's X-ray abdomen (Figure 5).

**Table 1:** Laboratory investigations performed at presentation (year 2018) and during follow-up ALT: alanine amino-transferase; AST: aspartate amino-transferase; HCO_3-_: derived bicarbonate levels from arterial blood gas analysis; iPTH: intact parathyroid hormone; pCO_2_: partial pressure of carbon dioxide; pO_2_: partial pressure of oxygen

Parameters	Year of follow-up					Reference range
-	2018	2019	2021	2022	2023	-
Hemoglobin (gm/dl)	12.5	12.1	12.3	-	12.9	13.5 to 17.5
Total leukocyte count (x10^3^/mm^3^)	5.6	4.5	5.1	-	5.6	4.0 to 11.0
Platelet count (x10^3^/mm^3^)	233	212	194	-	219	150 to 450
Blood urea nitrogen (mg/dl)	12	20	11	15	14	6 to 24
Creatinine (mg/dl)	0.8	1	0.7	0.8	0.7	0.7 to 1.3
Serum sodium (mEq/dl)	142	144	143	141	142	135 to 145
Serum potassium (mEq/dl)	2.1	2.7	1.9	3.1	3.7	3.5 to 5.2
Serum calcium (mg/dl)	8.6	8.3	7.9	8.2	8.9	8.5 to 10.3
Serum phosphorous (mg/dl)	4.3	4.1	-	4.1	4.5	2.8 to 4.5
Serum chloride (mEq/L)	116	-	-	109	-	96 to 106
Serum magnesium (mEq/l)	1.1	-	-	1.4	-	1.7 to 2.2
ALT (IU/ml)	23	21	21	22	23	7 to 56
AST (IU/ml)	19	21	23	18	19	8 to 33
Serum albumin (gm/dl)	4.1	-	-	4.3	-	3.4 to 5.4
Alkaline phosphatase (IU/l)	36	41	52	37	56	47 to 147
Vitamin D (ng/ml)	45	134	-	57	61	more than 20
iPTH (picogram/ml)	11.2	-	-	-	-	10 to 65
Arterial blood gas analysis	-					-
pH	7.27	7.35	7.31	7.37	7.41	7.35 to 7.45
pCO_2_ (mmHg)	44	45	44	42	41	35 to 45
pO_2 _(mmHg)	89	88	83	87	89	75 to 100
HCO_3-_ (mmol/l)	18.9	21.8	19.1	24.8	26.1	22 to 28
Urinary pH	8	7	8	7.5	6	4.6 to 8

**Table 2:** Urine analysis of study patient at presentation U: urinary

Parameters	Value	Reference range
Urinary pH	8	4.6 to 8
Urinary sodium (mmol/l)	41	40 to 220
Urinary potassium (mmol/l)	23	3.5 to 5
Urinary chloride (mmol/l)	28	98 to 107
Urine bicarbonate (mmol/l)	11	0 to 20
Spot urinary creatinine (mg/dl)	12	2o to 320
Urinary anion gap (UAG) ((U. sodium + U. potassium) - U. chloride) (mmol/l)	(41+23) - 28 = 36	less than 10
Fractional excretion of bicarbonate ((urine HCO₃⁻ × plasma creatinine/plasma HCO₃⁻ × urine creatinine) × 100%)	(11 × 0.8/18.9 × 12) × 100 = 3.8%	less than 5%

Based on the history, physical examination, and biochemical analysis, the diagnosis of rickets secondary to dRTA was made. Additional investigations were performed to determine the etiology of dRTA. The autoimmune panel excluded systemic lupus erythematosus and Sjögren syndrome, while abdominal ultrasound ruled out obstructive uropathy. There was no prior history indicative of exposure to drugs associated with dRTA. However, due to financial constraints, further investigations were not performed.

**Figure 6:**
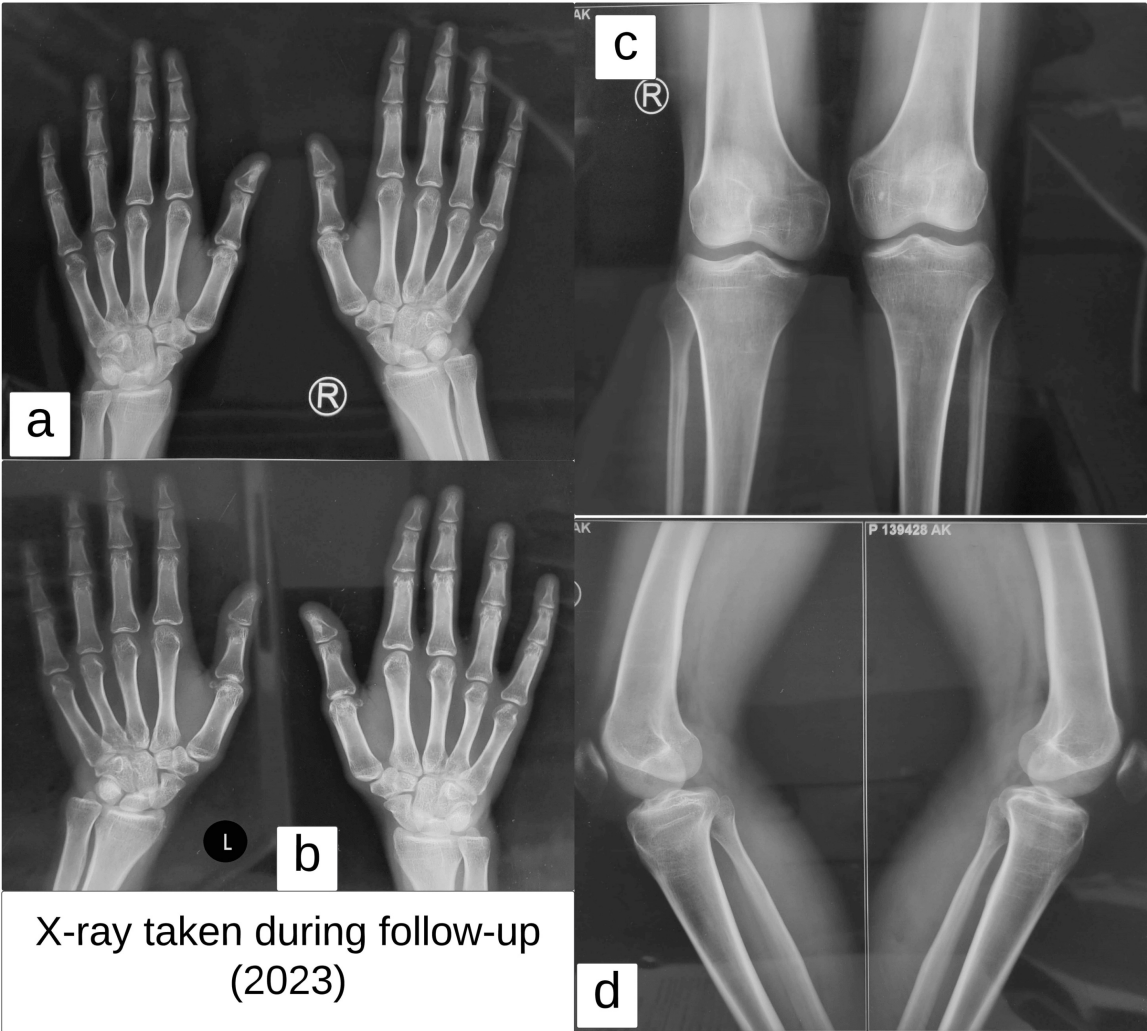
Follow up X-ray showing (a) and (b) X-ray hand and wrist; (c) and (d) X-ray bilateral lower limb showing marked improvement in the previously widened, frayed, and cupped metaphysis of long bones, with the meta-diaphyseal region appearing significantly more uniform, genu valgum showing partial correction (c) and (d), and there is also a significant increase in bone density compared to the previous X-ray (Figure 4)

Initially, in-hospital management was done, and the abnormal electrolytes were corrected. The patient was discharged with proper dietary advice and supplemented with oral potassium citrate and magnesium citrate solution. The patient was followed for eight to 12 weeks, and serial biochemical analysis was performed. The patient showed improvement in symptoms, and no episodes of flaccid paralysis were recorded during the follow-up. The patient showed improvement over time in weight and height and had catch-up growth in weight for age and height for the age (Figures 2, 3). A follow-up X-ray of the bilateral lower limbs and hand with wrists was carried which revealed an improvement of the previously widened, frayed, and cupped metaphysis of long bones, the meta-diaphyseal region appeared significantly more uniform, the genu valgum showed partial correction, and there was a significant increase in bone density as compared to previous studies (Figure 6).

## Discussion

**Figure 7:**
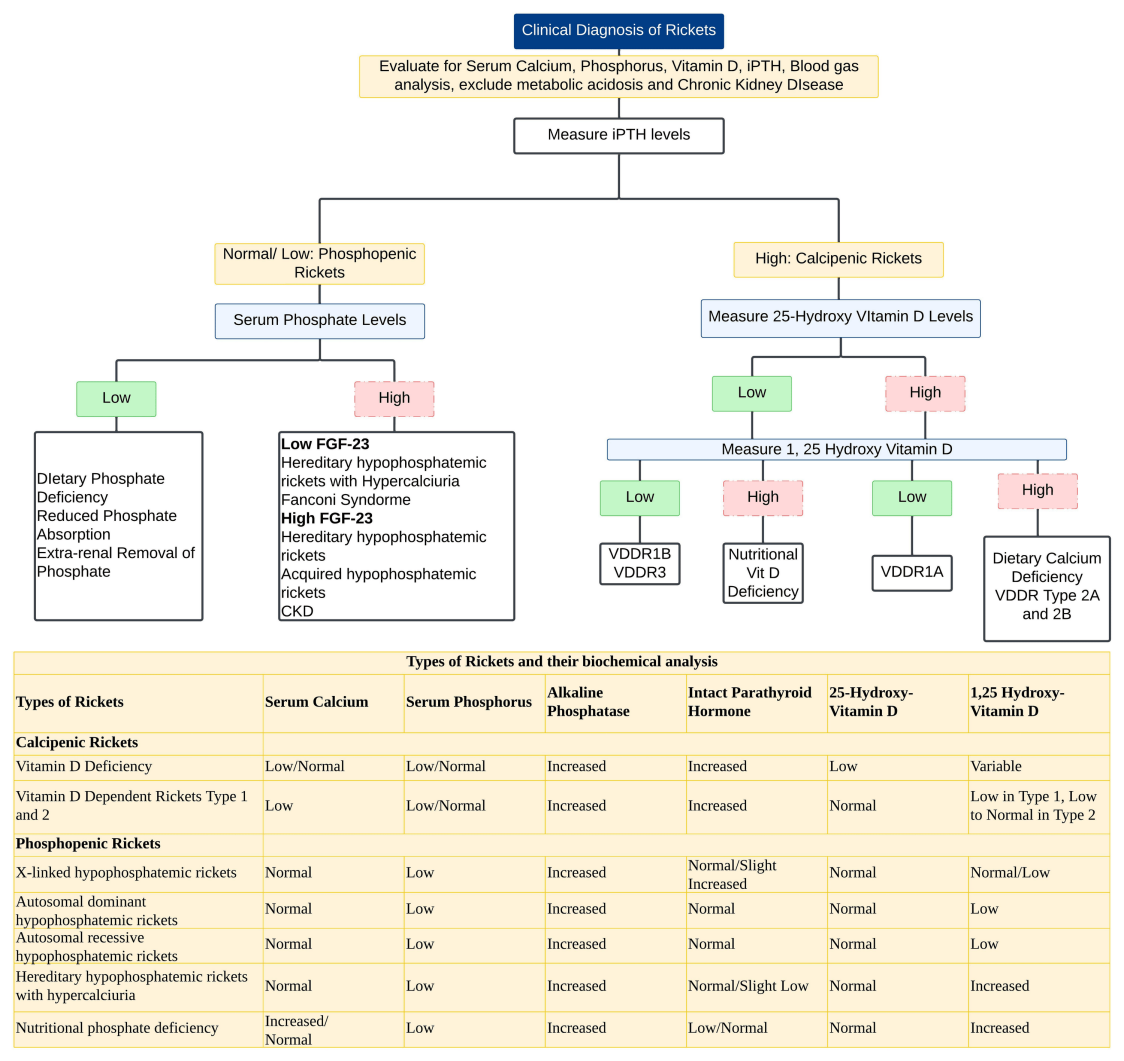
Approach to a patient with clinical suspicion of rickets FGF 23: fibroblast growth factor 23;  iPTH: intact parathyroid hormone; VDDR: vitamin D-dependent rickets The authors have used Lucid Chart, by *Lucid Software Inc.*, to illustrate the flowchart

**Figure 8:**
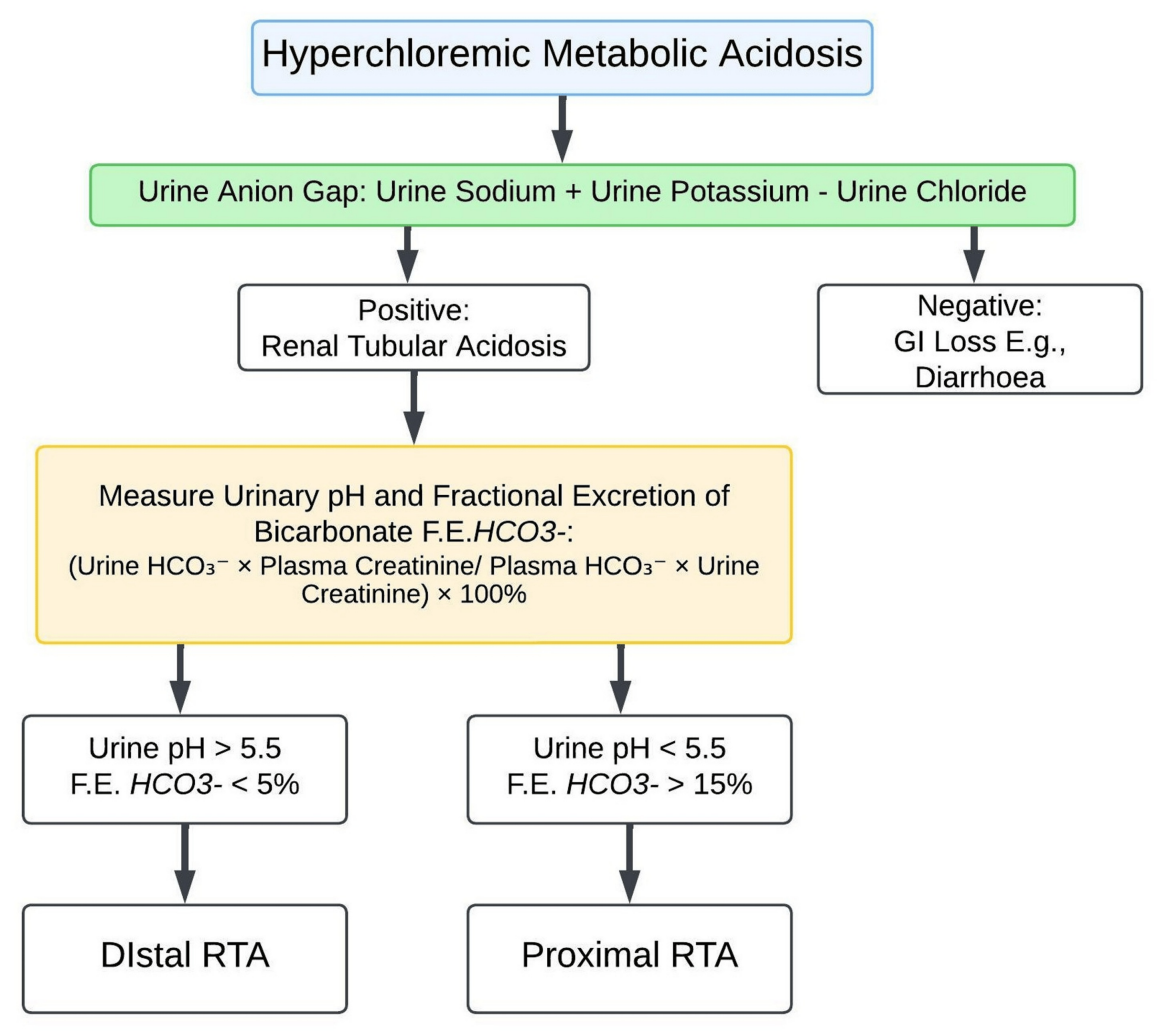
Approach to a patient with hyperchloremic metabolic acidosis [6] The authors have used Lucid Chart, by *Lucid Software Inc.*, to illustrate the flowchart

This case report highlights a rare etiology of rickets, dRTA, and emphasizes the need for a systematic and comprehensive approach in diagnosing a patient who presented with skeletal deformities and periodic weakness. While nutritional rickets due to vitamin D deficiency remains the most prevalent form, this case illustrates the importance of considering alternative diagnoses in patients with rickets and abnormal biochemical parameters. The classical approach to a patient with suspected rickets is shown in Figure 7 [1]. The presentation of bilateral genu valgum and short stature, along with intermittent muscle weakness and fatigue along with hyperchloremic metabolic acidosis, urinary alkalosis, and normal vitamin D levels pointed toward a systemic metabolic derangement. The approach to a patient with hyperchloremic metabolic acidosis is shown in Figure 8 [6].

RTA can be classified into four categories: type 1 (distal RTA), type 2 (proximal RTA), type 3 (mixed RTA), and type 4 [4]. Type 1 dRTA is caused by the impaired ability of the α intercalated cells of the kidney to eliminate hydrogen ions in urine. This results in systemic acidosis accompanied by an alkaline urine pH [4]. Type 2 proximal RTA occurs due to deficient bicarbonate absorption, resulting in excessive bicarbonate excretion in urine and systemic acidosis [4]. It can occur as an isolated renal tubular deficiency or a component of a widespread tubular disease referred to as Fanconi’s syndrome. Type 3 RTA (both proximal and distal or mixed) is uncommon and often occurs in an autosomal recessive disorder resulting from hereditary carbonic anhydrase deficiency [4]. Type 4 RTA results from hyporeninemic hypoaldosteronism, presenting as hyperkalemia and metabolic acidosis, often seen in diabetes and in cases of mild to moderate chronic glomerular insufficiency [4]. Type 1 RTA or dRTA is characterized by chronic metabolic acidosis which leads to hypophosphatemia, hypokalemia, vitamin D deficiency, and secondary hyperparathyroidism, which subsequently leads to rickets, osteomalacia, pathological fractures, and secondary osteoporosis [7]. The patient had biochemical findings suggestive of normal anion gap metabolic acidosis, hyperchloremia, hypokalemia, an elevated urinary anion gap, and low fractional excretion of bicarbonate which were diagnostic of dRTA. Radiological evidence, including metaphyseal widening, cupping, and nephrocalcinosis, further supported this diagnosis.

Chronic acidosis promotes calcium mobilization from the bone and impairs phosphate deposition, contributing to skeletal deformities [8]. In addition, hypercalciuria, a hallmark of dRTA, predisposes patients to nephrocalcinosis and nephrolithiasis which was observed in this case [9]. The association of these metabolic abnormalities along with progressive bone deformities underscores the systemic nature of the disorder and its significant impact on growth and development. The management of dRTA is aimed at correcting metabolic acidosis and associated electrolyte disturbances to halt the progression of skeletal and renal complications. Alkali therapy, in the form of potassium citrate, not only corrected the metabolic acidosis but also reduced hypercalciuria, which in turn mitigated the risk of further nephrocalcinosis. Over the five-year follow-up, the patient demonstrated significant clinical and radiological improvement, including partial correction of the genu valgum deformity and normalization of bone density. This highlights the efficacy of early diagnosis and timely intervention in preventing irreversible skeletal and renal damage.

This case underscores the importance of a comprehensive approach in pediatric patients presenting with rickets. Nonnutritional etiologies, such as renal tubular disorders, should be considered in cases with normal vitamin D levels or when conventional treatment fails to produce expected outcomes. Furthermore, the presence of nephrocalcinosis on imaging should prompt evaluation for underlying metabolic disorders, including dRTA. While autoimmune and genetic causes of dRTA were not identified in this case due to resource limitations, further investigation into the etiology of dRTA remains critical for tailoring management and predicting long-term outcomes.

## Conclusions

Recognizing rickets secondary to dRTA underscores the need for clinicians to consider less common etiologies of rickets to ensure timely and appropriate management. The observations in this report emphasize the role of long-term follow-up in monitoring the efficacy of treatment and disease progression. Regular assessment of biochemical parameters, growth metrics, and skeletal radiographs is essential to ensure sustained improvement and timely prevention of complications. Early diagnosis and tailored treatment can significantly improve the quality of life and long-term outcomes.
